# *In Vitro* Evaluation of Synergistic Essential Oils Combination for Enhanced Antifungal Activity against *Candida* spp.

**DOI:** 10.3390/life14060693

**Published:** 2024-05-28

**Authors:** Lukáš Hleba, Miroslava Hlebová, Ivana Charousová

**Affiliations:** 1 Institute of Biotechnology, Faculty of Biotechnology and Food Sciences, Slovak University of Agriculture in Nitra, Tr. A. Hlinku 2, 94976 Nitra, Slovakia; 2 Institute of Biology and Biotechnology, Faculty of Natural Sciences, University of SS. Cyril and Methodius, Nám. J. Herdu 2, SK-91701 Trnava, Slovakia; 3 Clinical Microbiology Laboratory, Unilabs Slovensko, s.r.o., J. Bellu 66, SK-03495 Likavka, Slovakia

**Keywords:** *Candida* spp., essential oils, synergy effect, antifungal activity, *in vitro*

## Abstract

In recent years, a significant number of infections have been attributed to non-albicidal *Candida* species (NAC), mainly due to the increasing resistance of NAC to antifungal agents. As only a few antifungal agents are available (azoles, echinocandins, polyenes, allylamines and nucleoside analogues), it is very important to look for possible alternatives to inhibit resistant fungi. One possibility could be essential oils (EOs), which have been shown to have significant antifungal and antibacterial activity. Therefore, in this study, the efficacy of 12 EOs and their combinations was evaluated against four yeasts of the genus *Candida* (*C. albicas*, *C. glabrata*, *C. tropicalis* and *C. parapsilosis*). GC-MS and GC-MS FID techniques were used for the chemical analysis of all EOs. VITEK-2XL was used to determine the antifungal susceptibility of the tested *Candida* spp. strains. The agar disc diffusion method was used for primary screening of the efficacy of the tested EOs. The broth dilution method was used to determine the minimum inhibitory concentrations (MICs) of the most potent EOs. After MIC cultivation, the minimum fungicidal concentration (MFC) was determined on Petri dishes (60 mm). The synergistic effect of combined EOs was evaluated using the checkerboard method and expressed as a fractional inhibitory concentration index (FICI). The results showed that ginger > ho-sho > absinth > dill > fennel > star anise > and cardamom were the most effective EOs. For all *Candida* species tested, the synergy was mainly observed in these combinations: ginger/fennel for *C. albicans* FICI 0.25 and *C. glabrata*, *C. tropicalis* and *C. parapsilosis* FICI 0.5 and absinth/fennel for *C. albicans* FICI 0.3125, *C. tropicalis* FICI 0.3125 and *C. parapsilosis* FICI 0.375. Our results suggest that the resistance of fungal pathogens to available antifungals could be reduced by combining appropriate EOs.

## 1. Introduction

Fungal infections, particularly those driven by *Candida* species, pose an escalating threat to global healthcare systems [1]. The rise of drug-resistant strains, coupled with the adaptability of these pathogens to diverse environments, underscores the critical need for innovative antifungal strategies. In this landscape, essential oils (EOs) derived from various plant species have garnered attention for their complex chemical compositions and inherent biological activities [2].

Situated at the intersection of environmental and medical sciences, this study explores the broader implications of EOs in combating *Candida* infections. *Candida*, ubiquitous in nature, has become a formidable public health concern, exacerbated by the emergence of drug-resistant strains such as *Candida auris*. This multidrug-resistant yeast species has rapidly spread globally, causing severe infections in healthcare settings and highlighting the urgent need for novel therapeutic interventions [3].

The identification of *Candida* strains, regardless of specific origins, is a pivotal step in comprehending the dynamic interactions between these fungi and their surroundings. Methods ranging from traditional culture-based approaches to advanced techniques like MALDI-TOF MS provide a taxonomy essential for contextualising subsequent investigations [4]. Simultaneously, the chemical profiling of EOs assumes significance. Gas chromatography-mass spectrometry (GC-MS) and gas chromatography with flame ionisation detector (GC-FID) techniques unravel the intricate compositions of these EOs. This chemical diversity not only enriches our understanding of EOs but also aligns with broader investigations into the potential therapeutic properties of plant-derived compounds [5]. Antifungal susceptibility testing, often utilising standardised systems like Vitek-2 XL, establishes a baseline for strains’ responses to conventional antifungals. The primary screening of EO activity, a prevalent approach in antifungal research, serves as an initial filter, identifying oils with promising antifungal properties [6]. Further exploration involves determining the minimum inhibitory concentration (MIC) and minimum fungicidal concentration (MFC) of selected EOs. These quantitative metrics provide nuanced insights into the concentrations required for inhibiting and eradicating *Candida* growth. Such investigations contribute not only to our understanding of essential oils’ therapeutic potential but also to the broader field of natural product research [7,8].

In the broader scientific context, this study aligns with the growing body of research emphasising the potential of natural compounds in combating fungal infections. EOs, with their intricate chemical profiles, serve as a reservoir of bioactive constituents, illustrating the untapped potential for developing effective antifungal agents [9].

The main objective of this study was to test four *Candida* species (*C. albicas*, *C. glabrata*, *C. tropicalis* and *C. parapsilosis*) for their susceptibility to 12 EOs, many of which have not been previously tested against *Candida* spp. This research contributes to the evolving narrative on antifungal strategies, emphasising the potential of EOs in combination. By integrating methodologies from various authors and addressing the ecological context of *Candida* strains, this study aims not only to advance our understanding but also to pave the way for the development of innovative and sustainable antifungal interventions. The urgency is underscored by the growing resistance observed in the formidable *Candida auris*, urging the exploration of alternative therapeutic avenues.

## 2. Materials and Methods

### 2.1. Yeast Strains’ Origin and Inoculum Preparation

The following strains of the genus *Candida* were used in this study: *Candida albicans*, *Candida glabrata* and *Candida tropicalis*. All strains used were previously obtained from polluted estuarine water. Strains of the genus *Candida* were identified using BD-Becton Dickinson Candida CHROMagar (Hi Media, Mumbai, India) and then by MALDI-TOF MS (Bruker Daltonics, Munich, Germany, Maldi Biotyper) using single colonies (48 h cultures) according to [10]. The spectra obtained were identified using Flex Control 3.4 software (Bruker Daltonics, Inc., Billerica, MA, USA) and MALDI Biotyper OC version 3.1 (Bruker Daltonics, Bremen, Germany). Prior to inoculum preparation, all tested species were cultured on Sabourad dextrose agar (SDA) (HiMedia, Mumbai, India) for 24–48 h in the dark at 30 ± 1 °C. The inoculum was prepared according to Hlebová et al. [11] to a final concentration of 2.5 × 10^5^ CFU/mL and the cell density was adjusted to the 0.5 McFarland turbidity standard 15–20 min before the analyses.

### 2.2. Essential Oils (EOs) and Their Chemical Analysis

Twelve EOs from different plant species, namely, ho-sho (*Cinnamomum camphora* Nees and Eberm var. Linaloolifera fujita), ginger (*Zingiber officinale* Rosco.), dill (*Anethum graveolens* L.), mint (*Mintha piperita* subsp. *Citrata* Ehrh.), juniper (fruit) (*Juniperum communis* L.), fennel (*Foeniculum vulgare* L.), cardamon (*Pelargonium graveolens* L.), myrrha (*Commiphora myrrha* Nees), absinth (*Artemisia absinthium* L.), star anise (*Illicium verum* Hook. f.), sweet flag (*Acorus calamus* L.), and tea tree (*Melaleuca alternifolia* L.) were used in this study. The EOs tested were supplied by Hanus (Nitra, Slovakia). Prior to analysis, the EOs were stored in closed, dark glass containers at 4 ± 1 °C. The chemical composition of all EOs used was analysed by gas chromatography-mass spectrometry (GC-MS) on an Agilent 7890A GC coupled to an Agilent-MSD5975C MS detector (Agilent Technologies, Palo Alto, CA, USA) with an HP-5MS column (30 m × 0.25 mm, 0. 25 m film thickness) and by gas chromatography with flame ionisation detector (GC-FID) techniques on Agilent 7890A (Agilent Technologies, PaloAl-to, CA, USA) with HP-5MS, measuring 30 m × 0.25 mm with 0.25 m film thickness, using the same method as the GC-MS analysis according to Bozik et al. [12]. The components of the EOs were identified by comparing their mass spectra with relative retention indices (RIs) according to the National Institute of Standards and Technology Library (NIST, USA) and available literature data by Adams [13], and authentic standards were used for the identification of (+)-α-Pinene, Camphene, (–)-β-Pinene, α-Phellandrene, p-Cymene, (R)-(+)-Limonene, Cineol, (–)-Linalool, Camphor, (+/–)-Citronellal, (–)-Borneol, (–)-Menthol, 4-Terpineol, Estragole, Nerol, (–)-Carvone, Geraniol, Bornyl acetate, Thymol, Eugenol, β-Caryophyllene, α-Caryophyllene, Pentadecane, Farnesene, Myristicin, and Caryophyllene oxide (Sigma-Aldrich, Munich, Germany). The chemical composition of the EOs was expressed as a percentage, and only those components equal to or greater than 1.00% were listed. All chromatograms were visualised by mMass version 5.5.0 software.

### 2.3. Vitek-2 XL Antifungal Susceptibility Test

To test the antifungal sensitivity, the identified isolates of *Candida* spp. were cultivated on BD-Becton Dickinson *Candida* CHROMagar and incubated at 37 °C for 48 h. After cultivation, 3 mL of sterile saline solution (aqueous 0.45% to 0.50% NaCl solution, pH 4.5 to 7.0) was transferred aseptically to the clear plastic (polystyrene) test tubes (12 mm × 75 mm) and the colonies of tested strains were resuspended in it. According to the manufacturer’s recommendations, the inoculum density was adjusted to 1.8–2.0 McFarland’s standard. The density of inoculums was measured on VITEK^®^ 2 DensiCHEK^®^ Plus (bioMérieux, Marcy d’Etoile, France). Subsequently, the prepared inoculums were transferred onto Vitek 2 AST-YS08 cards (bioMérieux, Marcy d’Etoile, France). Antifungal susceptibility testing (AST) was performed based on the recommended automated compact VITEK-2 XL with a yeast AST card. After inserting the cards with the inoculum, the incubation time was 10 to 27 h, based on the *Candida* spp. isolates’ growth rate in the control well without the antifungal drug. The cards were read automatically. A total of six antifungals were used for the strains of *C. albicans*, *C. tropicalis* and *C. parapsilosis*: fluconazole (0.5–64 µg/mL), voriconazole (0.12–8 µg/mL), micafungin (0.06–8 µg/mL), caspofungin (0.125–8 µg/mL), flucytosine (1–64 µg/mL) and amphotericin B (0.25–16 µg/mL). The *C. glabrata* strain used four antifungals in the same tested concentration range as previously: micafungin, caspofungin, flucytosine, and amphotericin B. Quality control for each new lot of cards was performed with different *Candida* spp., including *C. parapsilosis* (ATCC 22019) and *C. krusei* (ATCC 6258). The reference AST was performed according to EUCAST E.Def 7.3.2. [14,15]. The results were expressed as MICs in µg/mL and evaluated according to EUCAST [16]. Each isolate was tested three times.

### 2.4. Primary Screening of EO Antifungal Activity

First, a primary screening of the efficacy of the tested EOs was performed using the agar disc diffusion method according to Hlebova et al. [11], with small modifications. The test was performed on Petri dishes (150 mm) containing SDA medium (HiMedia, Mumbai, India), which were coated with 100 μL of the prepared inoculum on the surface of the SDA medium. Then, 13 empty filter paper discs (Ø 6.0 mm) (Oxoid Thermofisher S.p.A, Milan, Italy) impregnated with 50 μL of each tested EO were placed on the surface of the medium. The EOs were diluted in dimethyl sulfoxide (DMSO) (Sigma-Aldrich, Munich, Germany) (DMSO concentration ≤ 3%) and 0.5% Tween 80 (Sigma-Aldrich, Munich, Germany) to a final concentration of 512 μL/mL. The highest concentration was used to determine the sensitivity of the *Candida* spp. strains to the EOs and discs containing pure DMSO were used as a negative control (12 discs contained tested EOs and 1 disc with DMSO served as a control). The plates were incubated for 48 h at 37 ± 1 °C and then the inhibition zones were measured in two perpendicular planes using a digital scale. The zones formed by the tested *Candida* spp. strains during EO treatment were scored as follows:

- ≥12 mm (twice the disc size), the tested EXs/EOs had an inhibitory effect (IE) on yeast growth;
- ≤12 mm, the tested EXs/EOs had an average effect (AE) on yeast growth;
- <12 mm or 0 mm, the tested EXs/EOs had no effect (NE) on yeast growth [11].

### 2.5. Determination of Minimum Inhibitory Concentration (MIC) and Minimum Fungicidal Concentration (MFC) 

Only those EOs that inhibited the growth of the tested strains at a concentration of 512 µL/mL were selected for minimum inhibitory concentration (MIC) and minimum fungicidal concentration (MFC) testing (the EOs selected were those for which the inhibition zones formed by the yeasts were equal to or greater than 12 mm). MIC evaluation was performed on 96-well microtitre plates using the microdilution method as described by Hlebova et al. [11], with a slight modification in the plate arrangement. Selected diluted EOs were tested in the concentration range of 256 to 0.125 µL/mL. A total of 100 µL of Sabouraud’s broth medium (SBM) (HiMedia, Mumbai, India) was added to each well (except lines D1 to D12 and F1 to F12) of the microtitre plate (200 µL was added to the line from well G1 to G12). Each *Candida* strain was tested separately. Then, 100 µL of EO (wells A1 to C1) at a concentration of 256 µL/mL was added to column 1 and then transferred (100 µL) by the twofold dilution method from column 1 to column 12. The line from wells E1 to E12 served as a purity control for the EOs tested, the line from wells G1 to G12 served as a purity control for the medium and the line from wells H1 to H2 served as a positive control for the *Candida* spp. strains tested. The MIC lines (A to C) were separated by a control line (D1 to D6). EO purity control was also separated by a control line (F1 to F12). These lines were blank. After preparation of the microtitre plates, 100 µL of a *Candida* spp. inoculum was added to lines A to C and line H; 200 µL was the final volume in each well. The prepared microtitre plates were measured at 630 nm on the Opsys MRTM Microplate Reader to obtain the initial data. The microtitre plates were incubated for 48 h at a temperature of 37 ± 1 °C. After incubation, the microtitre plates were measured again and processed to determine the MIC for each EO tested.

The minimum fungicidal concentration (MFC) evaluation was performed on Petri dishes (60 mm) with SDA medium after cultivation according to Hlebová et al. [17]. From each well (wells containing individual concentrations of selected EOs with inoculum), 100 µL was transferred to PDs using a micropipette. The PDs were sealed with parafilm and cultured as previously described (48 h at 37 ± 1 °C). After cultivation, the MFC was determined as the concentration at which no growth of the tested yeast strain was observed.

### 2.6. Synergistic Effect of Tested EOs and Results Evaluation

The evaluation of the synergistic effect of the dual combination of EOs was performed on a 96-well microtitre plate using the checkerboard method as described by Hlebova et al. [17]. Based on the results of the MIC determinations, the EOs were divided into two groups: those with low potency (EOs with MIC values ranging from 64 to 16 µL/mL) and those with high potency (EOs with MIC values ranging from 8 to 0.125 µL/mL). These EOs were selected to evaluate their potential synergistic effect in combination (low potency EOs + high potency EOs). EOs with very poor or no potency (EOs with MIC values > 512 µL/mL or between 512 and 128 µL/mL) were excluded from this experiment. The selected EOs were tested starting from their lowest concentration obtained in the MIC assessment, which depended on the individual *Candida* strains. Low-potency EOs and high-potency EOs were tested on a microtitre plate in two replicates. Testing was performed in duplicate (4 replicates in total). Of the tested concentrations, those that were finally used (depending on the *Candida* species tested) were prepared separately for each EO in microtubes. For each *Candida* species, the following combinations and concentration ranges of EOs (high-potency EO/low-potency EO) were used, depending on the response of the species tested: ginger (1–0.03125 µL/mL), ho-sho and absinth (2–0.0625 µL/mL) and dill/fennel (4–0.125 µL/mL), cardamon and star anise (32–1 µL/mL) for *C. albicans*; ginger (4–0.125 µL/mL) and ho-sho (8–0.25 µL/mL)/absinth (32–1 µL/mL) and fennel (64–2 µL/mL) for *C. glabrata*; ho-sho and ginger (4–0.125 µL/mL), dill and absinth/fennel (8–0.25 µL/mL) and star anise (64–2 µL/mL) for *C. tropicalis* and ho-sho, ginger, dill and absinth/fennel (4–0.125 µL/mL) and star anise (64–2 µL/mL) for *C. parapsilosis.* Then, a two-fold dilution of each EO was made separately and the mixtures of high-potency EOs and low-potency EOs were formed in 96-well microtitre plates; the final volume of each well was 200 µL. After preparation, the microtitre plates were measured and cultured as described in Section 2.5. After data acquisition, the antifungal effect of EOs in combination was evaluated according to the fractional inhibitory concentration index (FICI) using the formula FICI = (FIC1/MIC1) + (FIC2/MIC2), where FIC1 and FIC2 represent the fractional inhibitory concentrations of the combined EOs and MIC1 and MIC2 represent the minimum inhibitory concentrations of the individual EOs tested. The obtained results were evaluated as follows: synergistic (FICI ≤ 0.5), partially synergistic (0.5 < FICI ≤ 0.75), no effect (0.75 < FICI ≤ 1.5) and antagonistic (FICI ≥ 2) [11,17].

### 2.7. Statistical Analysis 

All experiments in this study were performed in three independent replicates. The test for the potential synergistic activity of EOs was performed in 4 replicates. Data were processed using Microsoft Office Excel computer software, and Statgraphics Centurion XVI (version 16.1.11) software (one-way ANOVA and Tukey HSD 95% multiple range test; *p* ˂ 0.05) was used for the statistical analysis of the results. MIC_50_ (MIC at which 50% of microorganisms are inhibited) and MIC_90_ (MIC at which 90% of microorganisms are inhibited) results were evaluated using probit analysis.

## 3. Results and Discussion

### 3.1. Antifungal Susceptibility Testing of Candida spp. Strains by the Vitek-2 XL Test

In this study, the three strains of the genus *Candida* were tested for their antifungal resistance to commercially available drugs by the Vitek-2 XL antifungal susceptibility test. The results are summarised in Table 1. All tested strains of the genus *Candida* were susceptible to tested antimycotics.

### 3.2. Antifungal Susceptibility Testing of Candida spp. Strains to Studied EOs

To determine the sensitivity of the tested *Candida* spp. strains to the EOs, primary screening of their antifungal effect at the highest tested concentration of 512 μL/mL was performed. The zones of inhibition formed around the growth of yeast of the genus *Candida*, which were equal to or greater than 12 mm (compared with the disc size (6 mm)), indicated the efficacy of the EOs. The results are shown in Table 2.

Myrrh and juniper EOs were found to be the least effective, with myrrh EO having no effect on the yeast *C. glabrata* (IZ of 0.00 ± 0.00 mm). Similarly, EOs of mint citrata, sweet flag and tea tree only partially inhibited the growth of all species tested compared to the other tested EOs. The most effective EOs in the primary screening were ginger, ho-sho, absinth and dill. All these EOs had the greatest inhibitory effects on the growth of *C. albicans*, with the largest IZ diameters (28.23 ± 0.75 mm for ginger, 26.03 ± 0.25 mm for ho-sho, 23.77 ± 0.32 mm for absinth and 20.77 ± 0.25 mm for dill).

### 3.3. Minimum Inhibitory Concentration (MIC) and Minimum Fungicidal Concentration (MFC) Evaluations

Seven essential oils, namely, ho-sho, ginger, dill, fennel, cardamom, absinth and star anise, showed the ability to inhibit yeast growth at a concentration of 512 µL/mL (inhibition zone size equal to or greater than 12 mm). These EOs were selected for MIC and MFC determination using the microdilution method. The results for inhibitory activity were variable, but all EOs were able to either suppress or completely inhibit yeast growth (except *C. glabrata* treated with cardamom EO) (Table 3).

Based on the results of the MIC and MFC determinations, the inhibitory activity of the EOs depended mainly on the yeast species tested, but their efficacy can be reported in the following order: ginger > ho-sho > absinth > dill > fennel > star anise > cardamom. The lowest MIC_50_ or MIC_90_ value was predicted for *C. albicans* treated with ginger (MIC_50_ of 0.35 µL/mL and MIC_90_ of 1.43 µL/mL) and the highest MIC_50_ or MIC_90_ was predicted for *C. glabrata* treated with cardamom (MIC_50_ of 34.82 µL/mL and MIC_90_ of 94.70 µL/mL). Similarly, the lowest and highest fungicidal concentrations (MFCs) were recorded for these two EOs (ginger and cardamom EOs) against the same yeast species tested (an MFC of 2 µL/mL with the ginger EO treatment for *C. albicans* and an MFC of 256 µL/mL with the cardamom EO treatment for *C. glabrata*).

### 3.4. Chemical Analysis of Studied EOs

The chemical composition of the EOs used in this study and the authentic standards used are summarised in Table 4. The complete analyses (chromatograms) of the EOs can be found in the Supplementary Material, Figures S1–S12. According to our results, monoterpenes were the main constituents of all the plant EOs tested. Some EOs had a dominant presence of only one component, such as (−)-Linalool (97.80%) in ho-sho EO, (+)-α-pinene (41.50%) in juniper EO and trans-Anethole in fennel and star anise EOs (79.9% and 87.10%, respectively). (−)-Linalool was also present at the second highest level in mint EO (37.20%), but Geraniol (42.10%) was the most abundant. In ginger EO, (−)-Zingiberene (34.70%) was the most abundant, while dill EO was characterised by the highest content of (−)-Carvone (40.20%) and (R)-(+)-Limonene (37.10%). In cardamom EO, α-Terpineol acetate (43.7%) and Cineol (33.10%) were the major constituents. Benzofuran (33.40%) and Azulen-2-ol,1,4-dimethyl-7-(1-methylethyl)- (28.60%) were the most abundant components in myrrh EO. Absinth EO contained α-Terpinene (41.30%) as the major constituent and tea tree EO contained Menth-1-en-4-ol (40.50%) followed by γ-Terpinene (20.00%) in the lowest abundance.

### 3.5. Interaction Effect of Combined EOs Determination

The outcomes are presented in Table 5. For the most sensitive yeast tested, *C. albicans*, synergy was observed in three cases, with the most pronounced synergy being exhibited by the essential oil combinations ginger/star anise (FICI 0.1875), absinth/fennel (FICI 0.3125) and ho-sho/fennel (FICI 0.25), in that order. Two EO combinations, ho-sho/cardamom and dill/cardamon, were found to be antagonistic for this species. The other EOs were either partially synergistic or had no effect on *C. albicans* growth.

For the species *C. glabrata*, a synergistic effect of EOs was only observed for the combination of ginger/absinth EOs (FICI 0.515625). However, up to three combinations of EOs: ho-sho/dill, ho-sho/absinth and ginger/dill (FICI 2 for all combinations tested) had antagonistic effects on the growth of this species. For *C. tropicalis* and *C. parapsilosis*, the strongest synergistic effect was found for the combination of absinth/dill (FICI 0.3125 for *C. tropicalis* and FICI 0.375 for *C. parapsilosis*). No antagonistic effect of EOs was observed for these two species.

## 4. Discussion

Infections caused by opportunistic pathogenic fungi, especially species of the genus *Candida*, *Cryptococcus neoformans* and *Aspergillus fumigatus*, are a serious medical problem, especially in immunocompromised patients, and their incidence has been increasing significantly in recent years [18]. Few antifungal agents are available for treating systemic mycoses, which include mainly polyenes, allylamines, azoles, fluoropyrimidines and echinocandins, which have different mechanisms of action [19]. Concretely, amphotericin B, flucytosine and the azole derivatives fluconazole, itraconazole and ketoconazole are currently available. In our study, the resistance to antifungal drugs was not recorded in any of the tested species of the genus *Candida* (Table 1). However, these strains were not directly clinical isolates but species found in polluted estuarine water. Until now, the resistance between individual yeast species or strains has only been a serious problem with flucytosine. However, resistance among *Candida* spp. to orally administered azole derivatives was also observed [20], for example, in the case of species *C. auris*, which shows a reduced sensitivity to azoles, polyenes and echinocandins [21]. So, there is a concern that pathogens can develop resistance to all these agents. 

For this reason, there is an intense need to search for new compounds because the current therapeutic regimens are limited in terms of their toxicity and cost–effectiveness [22]. Therefore, there is an increasing need worldwide to reduce the use of synthetic substances as antimicrobial agents in the field of nutrition and the fight against various infections due to increasingly aggressive endogenous microorganisms that are resistant to their use [23,24]. Since antifungals can act synergistically with EOs, the combination of EOs with antifungals could be developed in the future for treatment against *Candida* spp. Therefore, the sensitivity of these yeast strains (*C. albicans*, *C. glabrata*, *C. tropicalis* and *C*. *parapsilosis*) to twelve EOs was further tested in this study. All EOs were first tested at a higher concentration (512 µL/mL) to verify their inhibitory activity against the test species. The results (Table 2) showed that the EOs with the highest inhibitory activity were ho-sho, ginger, absinth and dill [25]. The antifungal activity of ho-sho (*Cinnamonum*) EO has already been proven against *C. albicans*, *Sacharomyces cerevisiae*, *S. pombe* [25] and *Alternaria solani* [26]. The antifungal activity of ginger EO has also been proven against *C. albicans* [27] and *Aspergillus* species [17]. Obistioiu et al. [28] tested four EOs from some *Artemisia* spp. (*A. dracunculus*, *A. abrotanum*, *A. absinthium* and *A. vulgaris*) against *C. albicans*. Their results showed that EO from *A. absinthium* was the second most effective EO against *C. albicans*, with an IZ of 17 ± 1.4 mm. Taherkhani et al. [29] also found that EO from *A. absinthium* showed significant activity against *Candida albicans* (ATCC 5027). This is similar to our study, where this EO was the third most effective against all *Candida* species tested, with inhibition zones ranging from 18.03 to 23.77 mm. Furthermore, in this study, *C. albicans* was the most sensitive yeast to the action of these EOs (ho-sho, ginger, absinth and dill EO) when its mycelium formed the largest inhibition zones (IZs) around the discs. In contrast, *C. glabrata* was the most resistant to the action of all the EOs tested (including the most potent ones). Khosravi et al. [30] investigated the composition and anti-*Candida glabrata* activity of *Artemisia siberi* and *Origanum vulgare* EOs. Their results showed that all *C. glabrata* strains tested were sensitive to these EOs, but their activity depended on the concentration used. The authors Hrytsyk et al. [31] also tested the antifungal activity of *Artemisia* L. extracts using clinical strains of *Candida albicans* and *Candida tropicalis* that were resistant to polyene antibiotics, imidazoles and triazoles. The authors found similar significant fungicidal activity of *Artemisia* spp. herbal extract against *Candida tropicalis*. Likewise, myrrh and juniper EOs were among the least effective of the EOs tested against clinical isolates of *C. albicans* and *C. tropicalis* [32,33] as well as *C. glabrata* and *C. krusei* [34]. 

Eight essential oils were selected to test their efficacy at lower concentrations (256–0.125 µL/mL) in MIC and MFC evaluations. The best MIC_50_ and MIC_90_ values were predicted for *C. albicans* with ginger oil (0.35 µL/mL and 1.43 µL/mL) > absinth (0.57 and 1.56 µL/mL) > ho-sho (0.93 µL/mL and 1.97 µL/mL) > dill (1.39 µL/mL and 3. 29 µL/mL) > fennel (6.67 µL/mL and 13.75 µL/mL) > star anise (7.31 µL/mL and 13.08 µL/mL) > and cardamom (8.96 µL/mL and 16.80 µL/mL). In this study, *C. albicans* was also among the most sensitive of the yeasts tested. Similar results were obtained by López et al. [35], who tested ginger EO against different bacteria, microscopic filamentous fungi and yeasts. They found that *C. albicans* was the most sensitive to the action of this essential oil, with a MIC value of 0.25 mg/mL, and *C. tropicalis* and *C. glabrata* were the most resistant, with MIC values of 0.125 mg/mL and 0.75 mg/mL, respectively. Our results show that cardamon EO was the least potent EO. Better results with cardamom EO were obtained by Pattnaik et al. [36], who tested the antibacterial and antifungal activity of four EOs (cardamom, peppermint, cinnamon and orange) and found that cardamom essential oil was the second most effective against all microorganisms tested, with a MIC of 3.15 µL/mL for *C. albicans*. However, several authors describe its significant antibacterial effects [37,38]. In the work of Karameşe and Özgür [39], who tested 23 different EOs against several bacterial species as well as yeasts (*C. albicans*, *C. glabrata* and *C. parapsilosis*), cardamon EO appeared to be less effective, with MICs for bacteria and yeasts between 31.5 and 62.5 µg/mL. The different effects of the EOs may be explained by their different chemical compositions.

According to our results, among the EOs that were able to inhibit the growth of all the tested yeasts at the lowest MIC and MFC values were ginger, ho-sho, absinth and dill EOs. The chemical compositions of the EOs analysed in this work are in agreement with those of other authors. Ginger EO was characterised by a high content of (−)-zingiberene (37.40%). Authors López et al. [35] tested the antibacterial and antifungal activity of ginger EO and found that it was able to inhibit the growth of the three tested yeasts (*C. albicans*, *C. glabrata* and *C. tropicalis*) much more than nystatin and ketoconazole, despite the fact that their essential oil contained much lower levels of zingiberene (6.56%), α-farnesene (3.57%) and sesquiphellandrene (2.67%) than the essential oil tested in our study (zingiberene (37.40%), α-farnesene (12.50%) and sesquiphellandrene (13.10%)). This shows that the content of minor components is very important for the antifungal and antibacterial properties of EOs and can influence the major components. In the same way, the monoterpenes carvone and linalool, which constituted major parts of the dill (40.20%) and ho-sho (97.80%) EOs tested in this study, are characterised by significant antifungal activity, which has been confirmed by many authors [40,41,42,43]. Medeiros et al. [44] tested linalool both *in vitro* and *in silico* and found that it acts on fungal cells by disrupting their cell wall and plasma membrane by interacting with important enzymes involved in the biosynthesis of these fungal structures. With regard to carvone, authors Pina et al. [43] confirmed in their study that carvone and its derivatives ((R)-(–)-carvone and (S)-(+)-carvone) have significant antifungal and antibacterial effects. The authors Oosterhaven et al. [45] report that carvone acts by disturbing the metabolic energy status of the cell.

The use of fluorescence and scanning electron microscopy on yeast cells treated with EOs in a study by Alderees et al. [46] illustrated the fungicidal mechanisms of these EOs. Research indicates that polygodial and citral cause structural disruptions in cell membranes, elevate membrane permeability, and create channels or lesions in cytoplasmic membranes, resulting in the leakage of intracellular contents and subsequent cell death [47,48,49]. Yeast cell death has been linked to several mechanisms: lysis of the cell membrane, the formation of membrane pores, and the leakage of cell components.

Furthermore, studies have revealed that the presence of exogenous ergosterol reduces the antimicrobial efficacy of EOs against yeast, leading to higher minimum inhibitory concentrations (MICs). The interaction between ergosterol and EOs was confirmed through an ergosterol binding assay. This interaction can be described as the binding of EOs (and their bioactive components) to ergosterol, which inhibits or diminishes its vital role in the cell membrane. Ergosterol, the primary sterol in fungal cell membranes, is crucial for maintaining membrane rigidity, fluidity, and permeability, which are essential for the function of membrane-bound enzymes and transporters [50]. Additionally, the binding of bioactive compounds to ergosterol in yeast cell membranes compromises membrane integrity and fluidity, potentially forming microspores or channels that allow ions and cellular contents to leak, ultimately leading to cell death. Similar mechanisms of fungal cell death via ergosterol binding have been observed with different EOs and their bioactive compounds, including thymol from *Thymus vulgaris* L., carvacrol, geraniol, nerol, and *Coriandrum sativum* L. leaf oil [51,52,53,54].

The sorbitol osmotic protection assay was employed to examine the impact of EOs on yeast cell wall integrity by Alderees et al. [46]. Increased MICs in the presence of sorbitol (0.8 M) suggest an interaction between EOs and the fungal cell wall. However, no change in the MICs of EOs was observed when exogenous sorbitol was added to the test media, indicating that the EOs did not affect the yeast cell wall. These results align with other studies reporting unchanged MICs for geraniol, thymol, nerol, eugenol, menthol, and terpinen-4-ol in the presence of 0.8 M sorbitol [53,55,56,57].

In short, the action mechanisms of EOs are influenced by their chemical makeup and the positioning of one or more functional groups on their molecules [58].

The primary proposed mechanism is membrane damage [59]. The solubility of EOs in the phospholipid bilayer of cell membranes plays a significant role in their antimicrobial effects. Clove oil, for example, has been reported to decrease the amount of ergosterol, which is a key component of fungal cell membranes [60]. Additionally, terpenoids in EOs have been shown to disrupt enzymatic reactions involved in energy metabolism [61]. 

Based on the results of the MIC determinations, all EOs that inhibited the growth of the tested yeasts were classified as high-potency (those whose MIC values for individual yeasts were equal to or less than 8 µL/mL), low-potency (all those whose MIC values were equal to or less than 64 µL/mL) or ineffective (all those whose MIC values were equal to or greater than 128 µL/mL) EOs. The high- and low-potency EOs were then tested by the checkerboard method at concentrations dependent on the individual yeast species tested. The highest number of combinations was tested for *C. albicans* (12 combinations in total) and the lowest for *C. glabrata* (6 combinations in total). The most frequently observed synergistic effect was for combinations of ho-sho and absinth EOs with fennel essential oil for all *Candida* species tested (ho-sho/fennel for *C. albicans*, FICI value of 0. 25, and *C. glabrata*, *C. tropicalis* and *C. parapsilosis*, FICI value of 0.5; absinth/fennel for *C. albicans*, FICI value of 0.3125; *C. tropicalis*, FICI value of 0.3125; and *C. parapsilosis*, FICI value of 0.375). Tomazoni et al. [62] describe the Linalool-rich ho-sho EO (98.8%) as significantly effective, with 100% growth inhibition of the tomato pathogen *Stemphylium solani* Weber. Similarly, fennel EO had a similar effect on this fungal species (growth inhibition of 99.4%) but achieved much higher MIC values (2.5 μL/mL) compared to our study (in our case, minimum MIC_50_ of 6.67 μL/mL and maximum MIC_90_ of 34.58 μL/mL). However, these authors did not test the combination of these EOs. In our case, their combination showed a synergistic effect on growth inhibition for all the species tested. These EOs interacted quite strongly, with the initial MIC values being reduced by at least half when they were combined. It is therefore likely that linalool enhances the efficacy of the other components of EOs.

In our study, absinth EO was also found to have a synergistic effect on the three yeast species tested in combination with fennel EO. Even when used alone, this EO is known to have significant antifungal properties [28,29,31]. Moussii et al. [63] report its synergistic effect with lavender and rosemary EOs, with great inhibitory potential on the growth of microorganisms. However, they mainly studied bacteria. The synergistic effect of the main components in absinth (α-Terpinene) and fennel EOs (tras-Anethol) in our study was also tested by the author Pavela [64] (in total, he tested up to 435 combinations of different components of EOs), who found that the combination of these two components, as well as many others, achieved the highest synergistic effect. However, EOs are currently being tested in combination with antibacterial drugs and have been shown to enhance their efficacy. For example, Bekka-Hadji et al. [65] found that a combination of absinth EO with cefoxitin had stronger antibacterial effects than when they were used separately. Many other authors have reported that a combination of EOs and an antimicrobial agent increases their efficacy and that their mutual use could lead to overcoming antibiotic or antifungal resistance in bacteria, microscopic filamentous fungi or yeasts.

EOs hold significant potential in clinical medicine for treating oral candidiasis and various skin diseases due to their broad-spectrum antimicrobial properties. These oils function primarily by disrupting cell membranes, which is particularly effective against fungi [66]. For instance, clove oil has been shown to reduce the quantity of ergosterol, a crucial component of fungal cell membranes, thereby compromising the integrity and functionality of the membrane and leading to cell death [46].

In the context of oral health, EOs such as clove oil [67], tea tree oil [68], and thyme oil [69] have been researched for their efficacy in preventing and treating oral candidiasis. Their ability to penetrate the biofilm and disrupt the fungal cell membrane makes them effective against *Candida* species, which are often resistant to conventional antifungal treatments [70].

For skin diseases, EOs can be applied topically to treat fungal infections like athlete’s foot, ringworm and other dermatophyte infections [66]. Oils such as tea tree oil [71], lavender oil [72] and eucalyptus oil [73] have demonstrated antifungal activity that can help clear infections and promote skin healing. The terpenoids and other active compounds in these oils interfere with fungal cells’ energy metabolism and enzymatic reactions, further enhancing their antifungal effects [74].

Moreover, the anti-inflammatory properties of many EOs can help reduce the symptoms associated with skin infections, such as redness, itching and swelling, thereby providing symptomatic relief alongside their antifungal actions [75]. Their use in clinical settings is supported by their natural origin and the growing concern over antibiotic and antifungal resistance, making EOs a valuable alternative or complementary treatment option in managing fungal infections of the mouth and skin.

The main potential of EOs lies in their antimicrobial, anti-inflammatory and antifungal properties. These characteristics make them valuable for treating infections, reducing inflammation and promoting healing. EOs can be used in clinical medicine for managing oral candidiasis, skin diseases and respiratory infections and even as natural preservatives in food and cosmetic products due to their ability to disrupt microbial cell membranes and inhibit growth.

## 5. Conclusions

This study tested the potential synergistic effect of 12 EOs against four species of *Candida* yeasts (*C. albicans*, *C. glabrata*, *C. tropicalis* and *C. parapsilosis*). The EOs that were among the most effective (achieving the lowest MIC and MFC values) were ginger, ho-sho, absinth, dill, fennel, star anise and cardamom. Chemical analysis of these potent EOs showed that they were particularly rich in (–)-Linalool (ho-sho), trans-Anethole (fennel, star anise), (–)-Zingiberene (ginger), (–)-Carvone (dill) and Cineol (cardamom). These EOs were therefore selected for synergy testing. For all *Candida* species tested, synergy was mainly observed in these combinations: ginger/fennel and absinth/fennel. But, according to our results, up to three combinations of tested EOs: ho-sho with dill and absinth and ginger with dill, had antagonistic effects on the growth of tested *Candida* species. However, the interpretation of synergism or antagonism in *in vitro* studies depends on the methodology used. Optimum conditions are established during *in vitro* testing, but, in a real organism, the results may be influenced by several factors. This is mainly because EOs are complex mixtures of different components and as these components react with each other, EOs will also interact with external factors such as pH, fats, water content and the presence of mucus or proteins. *In vivo* testing is therefore very important and may reveal further shortcomings or, on the contrary, advantages of the use of EOs in the fight against drug resistance. Since the methods used to evaluate the interactions of EOs vary widely, it is very important to develop a uniform, standardised method for testing EOs both *in vitro* and *in vivo*, which would provide a better knowledge of the mechanism of synergy of individual components of EOs, combinations of EOs or combinations of EOs or their components with drugs. Our results also show that it is very important to choose suitable EOs whose individual components are influenced by positive effects (as in the case of Linalool and trans-Anethole or Linalool and α-Terpinene), but the minor components of EOs should not be ignored either as they may have clinical significance (for example, they may increase the bioactivity of drugs). If we have more knowledge about the mechanism of the combination of EOs with antifungal substances, we could use them to increase the effectiveness of the drugs or reduce the dosage used, which would also prevent the emergence of resistant strains.

## Figures and Tables

**Table 1:** Tested *Candida* spp. strains and their sensitivity to antifungal drugs and obtained MICs (µg/mL) tested by Vitek-2 XL.

Antifungals	Tested Fungi							
	*Candida albicans*		*Candida glabrata*		*Candida tropicalis*		*Candida parapsilosis*	
	MIC	Category ^a^	MIC	Category ^a^	MIC	Category ^a^	MIC	Category ^a^
Fluconazole	0.5	S	–	–	0.25	S	0.25	S
Voriconazole	0.12	S	–	–	0.25	S	0.25	S
Caspofungin	0.12	S	0.03	S	0.12	S	0.12	S
Micofungin	0.06	S	0.03	S	0.06	S	0.06	S
Flucytosine	1	S	0.5	S	1	S	1	S
Amphotericin B	0.25	S	0.25	S	0.25	S	0.25	S

Note: MIC—minimum inhibitory concentration, ^a^—EUCAST breakpoints were used to categorise results into susceptibility categories, S—susceptible, standard dosing regimen.

**Table 2:** Means of inhibition zones (in mm ± SD) of tested *Candida* strains (3 repetitions were used for each strain (*n* = 3)) under treatment with EOs.

EOs	Tested Yeast			
	*C. albicans*	*C. glabrata*	*C. tropicalis*	*C. parapsilosis*
	Means of Inhibition Zone (IZ) Diameters (mm) ± SD			
Ho-Sho	26.03 ^g^ ± 0.25	22.37 ^h^ ± 0.55	25.57 ^i^ ± 0.67	25.57 ^h^ ± 0.51
Ginger	28.23 ^h^ ± 0.75	22.17 ^h^ ± 1.26	26.13 ^i^ ± 0.15	27.50 ^i^ ± 0.50
Dill	20.77 ^e^ ± 0.25	15.47 ^f^ ± 0.42	20.27 ^g^ ± 0.64	20.47 ^f^ ± 0.55
Mint citrata	11.27 ^c^ ± 0.87	8.00 ^c^ ± 0.92	9.80 ^d^ ± 0.26	10.23 ^c^ ± 0.87
Juniper	3.37 ^a^ ± 1.00	0.67 ^a^ ± 1.15	1.80 ^a^ ± 0.26	2.80 ^a^ ± 0.26
Fennel	16.70 ^d^ ± 0.44	14.27 ^ef^ ± 0.31	15.67 ^f^ ± 0.49	15.73 ^e^ ± 0.38
Cardamon	15.40 ^d^ ± 0.60	12.33 ^d^ ± 0.21	13.33 ^e^ ± 0.42	13.43 ^d^ ± 0.49
Myrrh	5.67 ^b^ ± 0.80	0.00 ^a^ ± 0.00	3.83 ^b^ ± 0.21	3.47 ^a^ ± 0.15
Absinth	23.77 ^f^ ± 0.32	18.03 ^g^ ± 0.25	22.20 ^h^ ± 0.53	22.60 ^g^ ± 0.53
Star anise	16.87 ^d^ ± 0.15	13.43 ^de^ ± 0.67	15.23 ^f^ ± 0.75	14.17 ^d^ ± 0.47
Sweet flag	10.73 ^c^ ± 0.64	5.80 ^b^ ± 0.26	6.63 ^c^ ± 0.21	7.63 ^b^ ± 0.55
Tea tree	10.70 ^c^ ± 0.79	7.77 ^c^ ± 0.25	9.77 ^d^ ± 0.25	10.30 ^c^ ± 0.62

Note: SD—standard deviation; data in the column followed by different letters were significantly different in 95.0% Tukey HSD test, *p* < 0.05.

**Table 3:** Minimum inhibitory concentrations (MICs) (MIC_50_—minimum inhibitory concentration at which 50% of microorganisms are inhibited; MIC_90_—minimum inhibitory concentration at which 90% of microorganisms are inhibited) and minimum fungicidal concentrations (MFCs) of selected EOs for tested *Candida* spp. (*n* = 9) evaluated by probit analysis.

Tested Fungi	MIC/MFC (µL/mL)		Tested Essential Oils						
			Ho-Sho	Ginger	Dill	Fennel	Cardamon	Absinth	Star anise
*C. albicans*	MIC	MIC_50_	0.93	0.35	1.39	6.67	8.96	0.57	7.31
		MIC_90_	1.97	1.43	3.29	13.75	16.80	1.56	13.08
	MFC		4	2	8	64	64	4	64
*C. glabrata*	MIC	MIC_50_	3.38	1.60	5.67	12.33	34.82	5.98	28.68
		MIC_90_	5.78	2.79	10.66	24.58	94.70	11.16	52.13
	MFC		16	16	32	128	512	64	256
*C. tropicalis*	MIC	MIC_50_	1.69	1.48	1.96	13.90	23.42	1.80	12.87
		MIC_90_	3.14	3.14	4.83	34.58	53.93	3.94	25.57
	MFC		8	8	16	128	256	16	128
*C. parapsilosis*	MIC	MIC_50_	1.65	0.58	1.53	10.34	19.13	1.01	11.74
		MIC_90_	3.20	1.68	2.68	24.25	44.03	2.15	22.72
	MFC		8	4	8	128	256	8	128

Note: Results of minimum inhibitory concentrations (MICs) and minimum fungicidal concentrations (MFCs) for tested EOs are expressed as µL/mL.

**Table 4:** Chemical composition (in %) of essential oils used in this study, determined by GC-MS and quantified by GC-FID techniques.

RI ^b^		Component	HS *^c^	G	D	M	J	F	C	My	A	SA	SF	TT
940	^a^	(+)-α-Pinene		1.10			41.50	2.10	2.10		2.50			2.40
954	^a^	Camphene		3.50										
978		Sabinene					8.90		2.40					
980	^a^	(–)-β-Pinene					4.70							
993	^a^	β-Myrcene				1.00	6.90				1.00			
1006	^a^	α-Phellandrene			9.40						2.50			
1013		(1S)-(+)-3-Carene					1.20							
1020	^a^	α–Terpinene									41.30			9.70
1029	^a^	p-Cymene			2.90		2.80		1.20					5.60
1033	^a^	(R)-(+)-Limonene		2.80	37.10	1.10	4.70	5.10	2.60					1.70
1035	^a^	Cineol							33.10		2.80		1.10	3.20
1063	^a^	γ-Terpinene					1.90							20.00
1091	^a^	Terpinolene					1.40	4.50	2.40					3.50
1101	^a^	(–)-Linalool	97.80			37.20			2.10		2.10	1.00		
1158	^a^	(+/–)-citronellal											1.30	
1177	^a^	(–)-Menthol				2.80								
1180	^a^	Menth-1-en-4-ol					5.40		1.20					40.50
1187		Anethofuran			4.60								87.60	
1192	^a^	4-Terpineol				1.30								2.90
1197		D-Dihydrocarvone			1.30									
1199		4-Allylanisole						4.40				5.30		
1205		4-Carvomenthenol			1.00									
1237		Asaron											5.40	
1247	^a^	(–)-Carvone			40.20									
1259	^a^	Geraniol				42.10		1.10	2.40			1.90		
1289		trans-Anethole						79.90				87.10		
1293	^a^	2-Undecanone									29.50			
1353	^a^	α-Terpineol acetate							43.70					
1368		Neryl acetate				2.30								
1386	^a^	Geranyl acetate				5.20								
1392		5-Methylindole					1.30			5.30				
1419	^a^	β-Caryophyllene					1.70				1.50			
1435		γ-Elemene								2.30				
1439		(+)-Aromadendrene												1.00
1454		α-Humulene					1.30							
1481		γ–Muurolene		1.30			1.80				1.50			
1485		α–Curcumene		13.20										
1490		β–Selinene		1.70							1.40			
1494		(+)-Ledene												1.30
1497		(–)-Zingiberene		34.70										
1498		Benzofuran								33.40				
1510	^a^	A-Farnesene		12.50										
1515		Benzene, (2-ethyl-4-methyl-1,3-pentadienyl)-, (E)-									1.20			
1524	^a^	Myristicin												1.40
1526		Sesquiphellandrene		13.10			2.00							
1557		Elixene					1.00			1.60				
1579	^a^	Caryophyllene oxide									1.00			
1628		Azulen-2-ol,1,4-dimethyl-7-(1-methylethyl)-								28.60				
1634		4,4′-Dimethyl-2,2′-dimethylenebicyclohexyl-3,3′-diene								9.40				
1678		(+)-Helminthogermacrene								1.20				
1680		(E)-foeniculin										1.10		
1721		(–)-Parthenolide								3.90				
1890		Acetic acid								1.50				
		total	97.80	83.90	96.50	93.00	88.50	97.10	93.20	87.20	88.30	96.40	95.40	93.20

Note: ^a^—identification confirmed by co–injection of authentic standard, ^b^ RI—identification based on Kovat’s retention indices (HP-5MS capillary column) and mass spectra, ^c^—relative proportions calculated in % by dividing individual peak area by total area of all peaks, *—HS—ho-sho, G—ginger, D—dill, M—mint, J—juniper, F—fennel, C—cardamon, My—myrrha, A—absinth, SA—star anise, SF—sweet flag, TT—tea tree.

**Table 5:** Synergistic, antagonistic or no effect of combined EOs (_HP_EOs/_LP_EOs—high-potency essential oils/low-potency essential oils), their MIC (minimum inhibitory concentration), FIC (fractional inhibitory concentration) and FICI (fractional inhibition concentration index [FIC(EO_HP_) + FIC(EO_LP_)]) in growth inhibition of tested Candida species.

EO Combination _HP_EOs/_LP_EOs	Fungal Strain							
	*Candida albicans*							
	MIC * EO_HP_	MIC * EO_HP_ in Presence of EO_LP_	MIC * EO_LP_	MIC * EO_LP_ in Presence of EO_HP_	FIC of EO_HP_	FIC of EO_LP_	FICI	Outcome
ho-sho/fennel	**2**	**0.25**	32	**8**	**0.125**	**0.125**	**0.25**	**synergism**
ho-sho/cardamon	2	4		16	2	0.5	2.5	antagonism
ho-sho/star anise	2	2		8	1	0.25	1.25	no effect
ginger/fennel	1	0.125	32	16	0.125	0.5	0.625	partial synergism
ginger/cardamon	1	0.5		32	0.5	1	1.5	no effect
ginger/star anise	**1**	**0.0625**		**4**	**0.0625**	**0.125**	**0.1875**	**synergism**
dill/fennel	4	0.5	32	16	0.125	0.5	0.625	partial synergism
dill/cardamon	4	4		32	1	1	2	antagonism
dill/star anise	4	4		8	1	0.25	1.25	no effect
absinth/fennel	**2**	**0.125**	32	**8**	**0.0625**	**0.25**	**0.3125**	**synergism**
absinth/cardamon	2	2		4	1	0.125	1.125	no effect
absinth/star anise	2	2		16	1	0.5	1.5	
	*Candida glabrata*							
ho-sho/dill	8	8	16	16	1	1	2	antagonism
ho-sho/fennel	**8**	**2**	**64**	**16**	**0.25**	**0.25**	**0.5**	**synergism**
ho-sho/absinth	8	8	32	32	1	1	2	antagonism
ginger/dill	4	4	16	16	1	1	2	
ginger/fennel	4	4	64	4	1	0.25	1.25	no effect
ginger/absinth	**4**	**0.0625**	**32**	**16**	**0.015625**	**0.5**	**0.515625**	**synergism**
	*Candida tropicalis*							
ho-sho/fennel	**4**	**1**	64	**16**	**0.25**	**0.25**	**0.5**	**synergism**
ho-sho/star anise	4	4		16	1	0.25	1.25	no effect
ginger/fennel	8	8		16	1	0.25	1.25	
ginger/star anise	8	1		32	0.125	0.5	0.625	partial synergism
dill/fennel	8	1		32	0.125	0.5	0.625	
dill/star anise	8	8		16	1	0.25	1.25	no effect
absinth/fennel	**8**	**0.5**		**16**	**0.0625**	**0.25**	**0.3125**	**synergism**
absinth/star anise	8	8		32	1	0.5	1.5	no effect
	*Candida parapsilosis*							
ho-sho/fennel	**4**	**1**	64	**16**	**0.25**	**0.25**	**0.5**	**synergism**
ho-sho/star anise	4	4		16	1	0.25	1.25	no effect
ginger/fennel	4	4		16	1	0.25	1.25	
ginger/star anise	4	1		32	0.25	0.5	0.75	partial synergism
dill/fennel	4	0.5		32	0.125	0.5	0.625	
dill/star anise	4	4		16	1	0.25	1.25	no effect
absinth/fennel	**4**	**1**		**8**	**0.25**	**0.125**	**0.375**	**synergism**
absinth/star anise	4	4		32	1	0.5	1.5	no effect

Note: * results of the minimum inhibitory concentrations (MICs) for EO_HP_ and EO_LP_ are expressed as µL/mL, Synergism in the Table 5 is marked in bold.

## Data Availability

The original contributions presented in this study are included in the article/Supplementary Material; further inquiries can be directed to the corresponding author/s.

## Supplementary Materials

The following supporting information can be downloaded at https://www.mdpi.com/article/10.3390/life14060693/s1: Figures S1: GC-MS FID chromatographic characteristics of ho-sho (*Cinnamomum camphora* Nees and Eberm var. Linaloolifera fujita) composition visualized in mMass software; S2: GC-MS FID chromatographic characteristics of ginger (*Zingiber officinale* Rosco.) composition visualized in mMass software; S3: GC-MS FID chromatographic characteristics of dill (*Anethum graveolens* L.) composition visualized in mMass software; S4: GC-MS FID chromatographic characteristics of mint (*Mintha piperita* subsp. *Citrata* Ehrh.) composition visualized in mMass software; S5: GC-MS FID chromatographic characteristics of juniper (fruit) *Juniperum communis* L.) composition visualized in mMass software; S6: GC-MS FID chromatographic characteristics of fennel (*Foeniculum vulgare* L.) composition visualized in mMass software; S7: GC-MS FID chromatographic characteristics of cardamon (*Pelargonium graveolens* L.) composition visualized in mMass software; S8: GC-MS FID chromatographic characteristics of myrrha (*Commiphora myrrha* Nees) composition visualized in mMass software; S9: GC-MS FID chromatographic characteristics of absinth (*Artemisia absinthium* L.) composition visualized in mMass software; S10: GC-MS FID chromatographic characteristics of star anise (*Illicium verum* Hook. f.) composition visualized in mMass software; S11: GC-MS FID chromatographic characteristics of sweet flag (*Acorus calamus* L.) composition visualized in mMass software; S12: GC-MS FID chromatographic characteristics of tea tree (*Melaleuca alternifolia* L.) composition visualized in mMass software.
