# Supplementary material for: In Vitro Evaluation of Synergistic Essential Oils Combination for Enhanced Antifungal Activity against Candida spp
Source: Life (Basel). 2024 May 28;14(6):693. doi: 10.3390/life14060693 (PMC11204509; doi:10.3390/life14060693)
Supplement: Supplementary file 1 [file life-14-00693-s001.zip › life-2997292-supplementary.pdf]

Article

The *In Vitro* Evaluation of Potential Synergistic activity of Essential oils and their Antifungal effects in combinations against *Candida* spp.

Lukáš Hleba [1]*, Miroslava Hlebová [2] and Ivana Charousová [3]

Correspondences: Lukáš Hleba (Lukas.hleba@uniag.sk), Slovak University of Agriculture in Nitra, Institute of Food Science, Faculty of Biotechnology and Food Sciences, Tr. A. Hlinku 2, 94976, Nitra, Slovak Republic.

Supplementary material

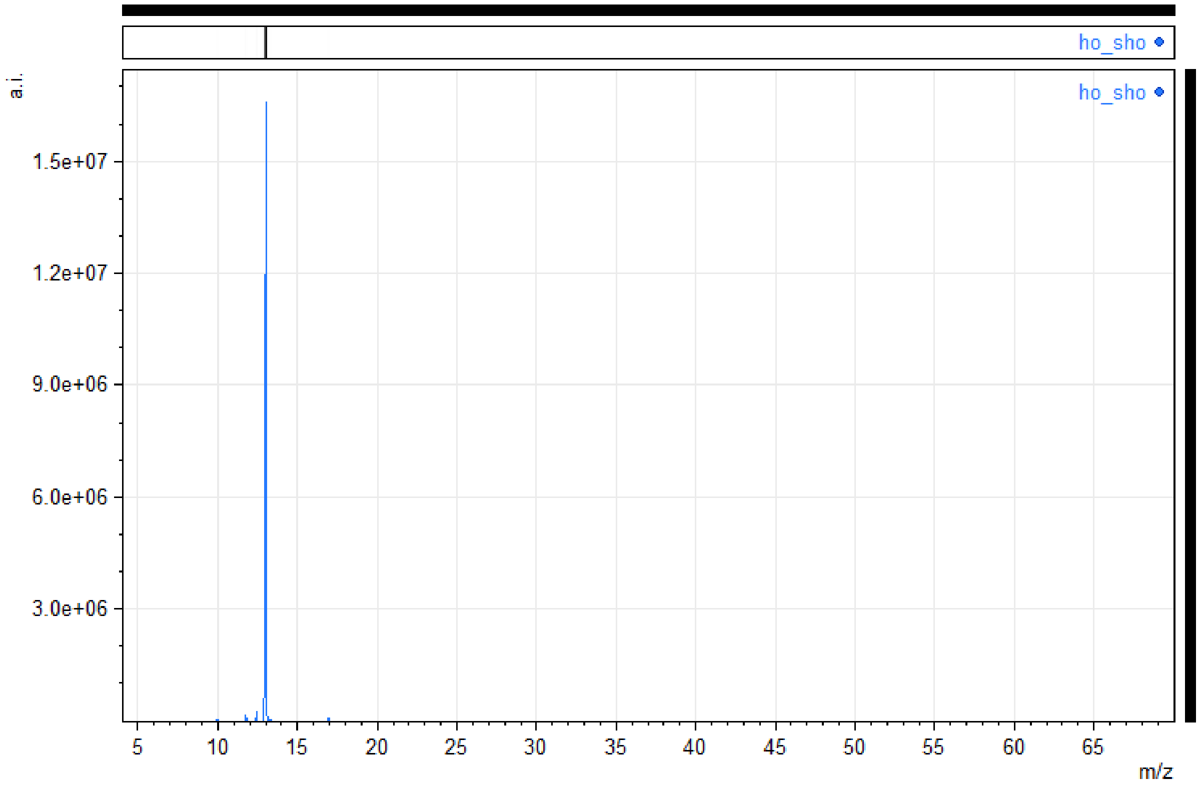

Figure S1. GC-MS FID chromatographic characteristics of ho-sho (*Cinnamomum camphora* Nees and Eberm var. Linaloolifera fujita) composition visualized in mMass software.

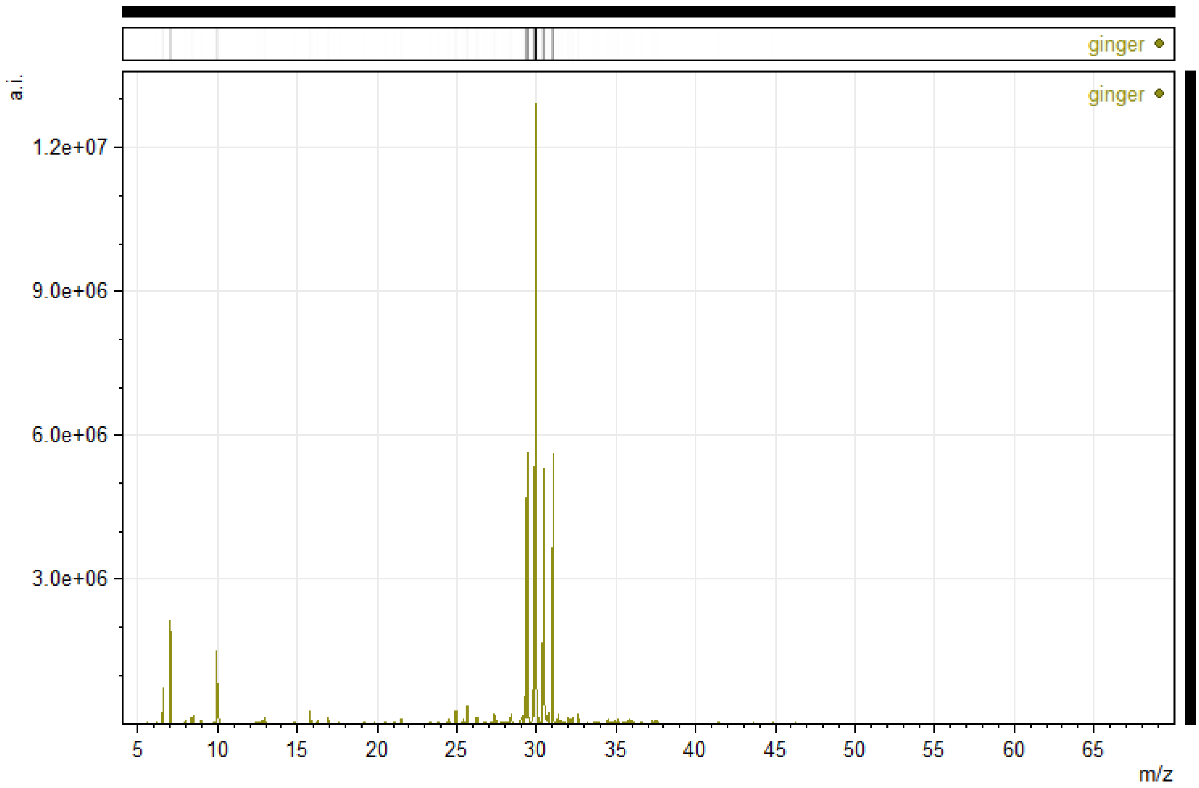

Figure S2. GC-MS FID chromatographic characteristics of ginger (*Zingiber officinale* Rosco.) composition visualized in mMass software.

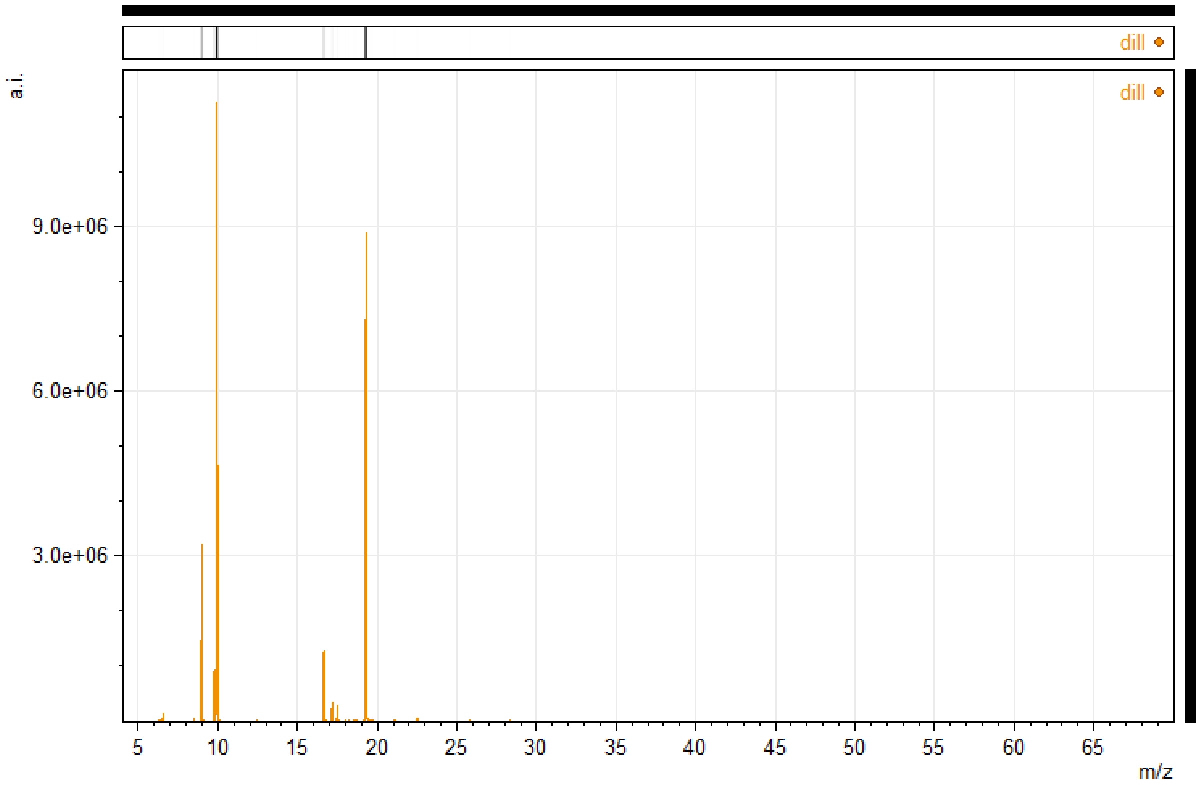

Figure S3. GC-MS FID chromatographic characteristics of dill (*Anethum graveolens* L.) composition visualized in mMass software.

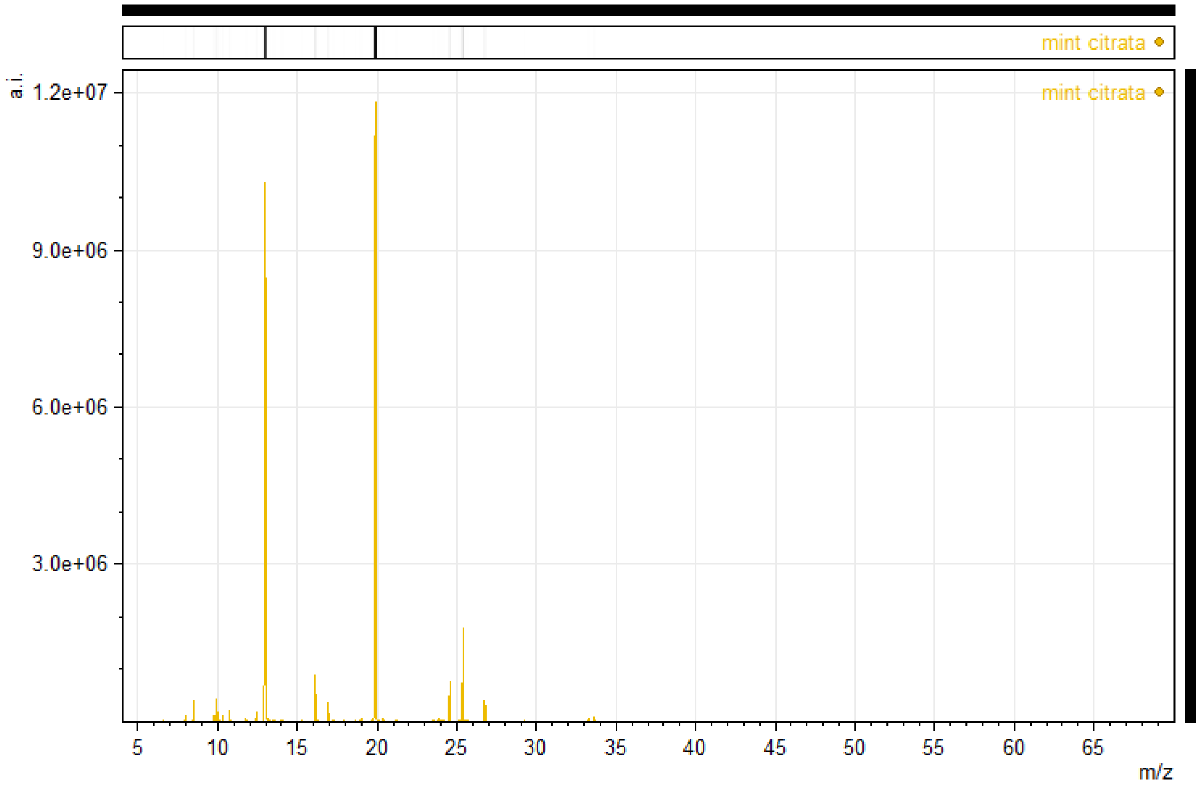

Figure S4. GC-MS FID chromatographic characteristics of mint (*Mintha piperita* subsp. *Citrata* Ehrh.) composition visualized in mMass software.

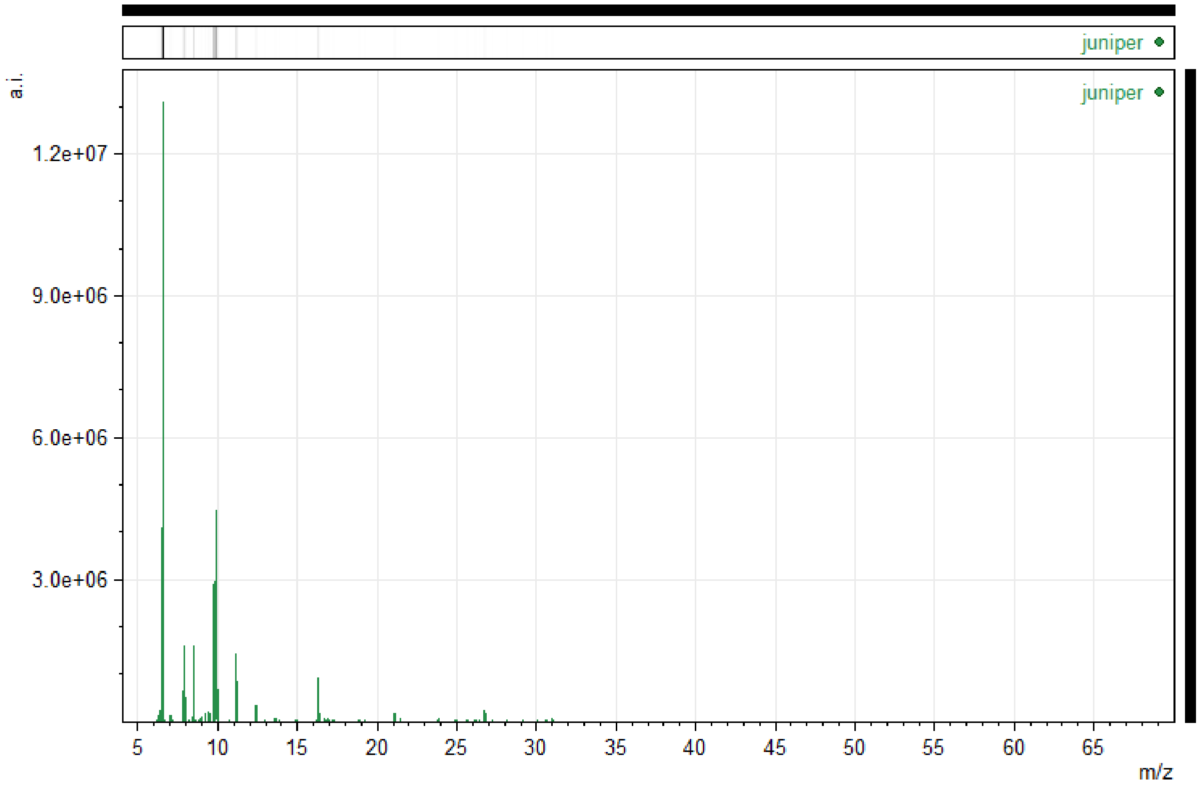

Figure S5. GC-MS FID chromatographic characteristics of juniper (fruit) *Juniperum communis* L.) composition visualized in mMass software.

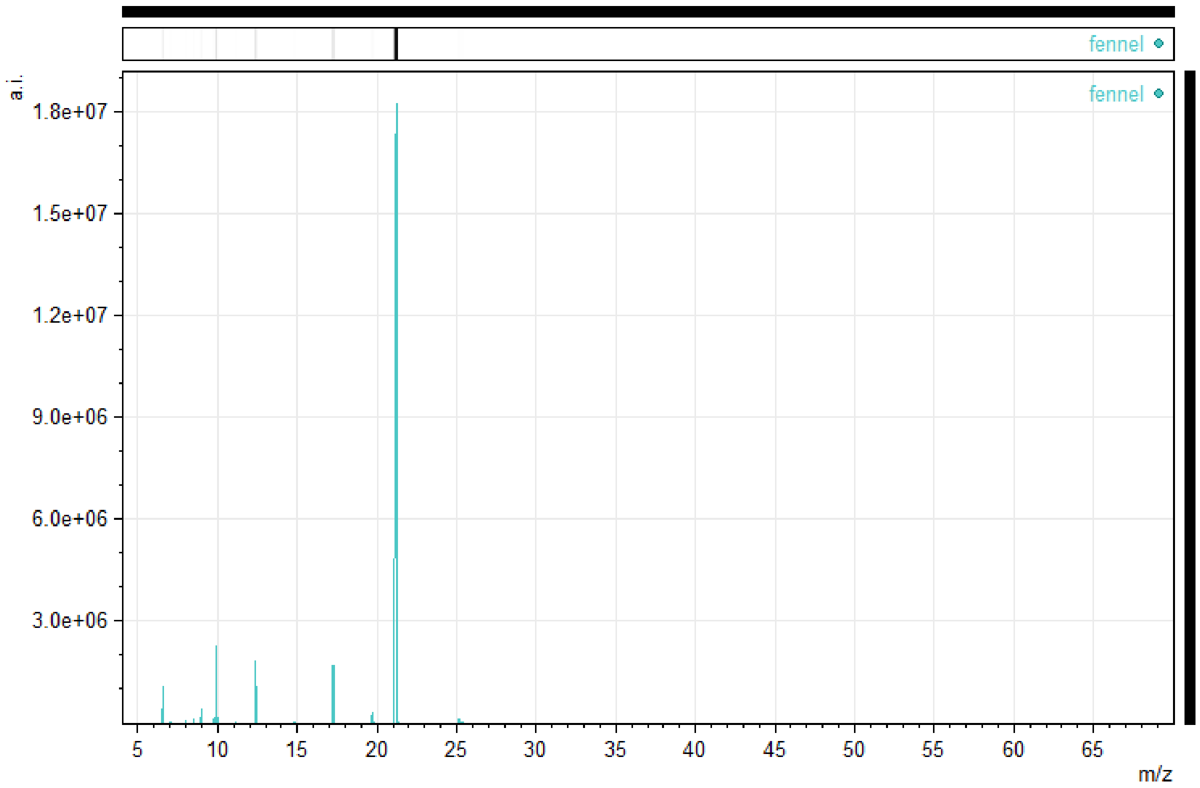

Figure S6. GC-MS FID chromatographic characteristics of fennel (*Foeniculum vulgare* L.) composition visualized in mMass software.

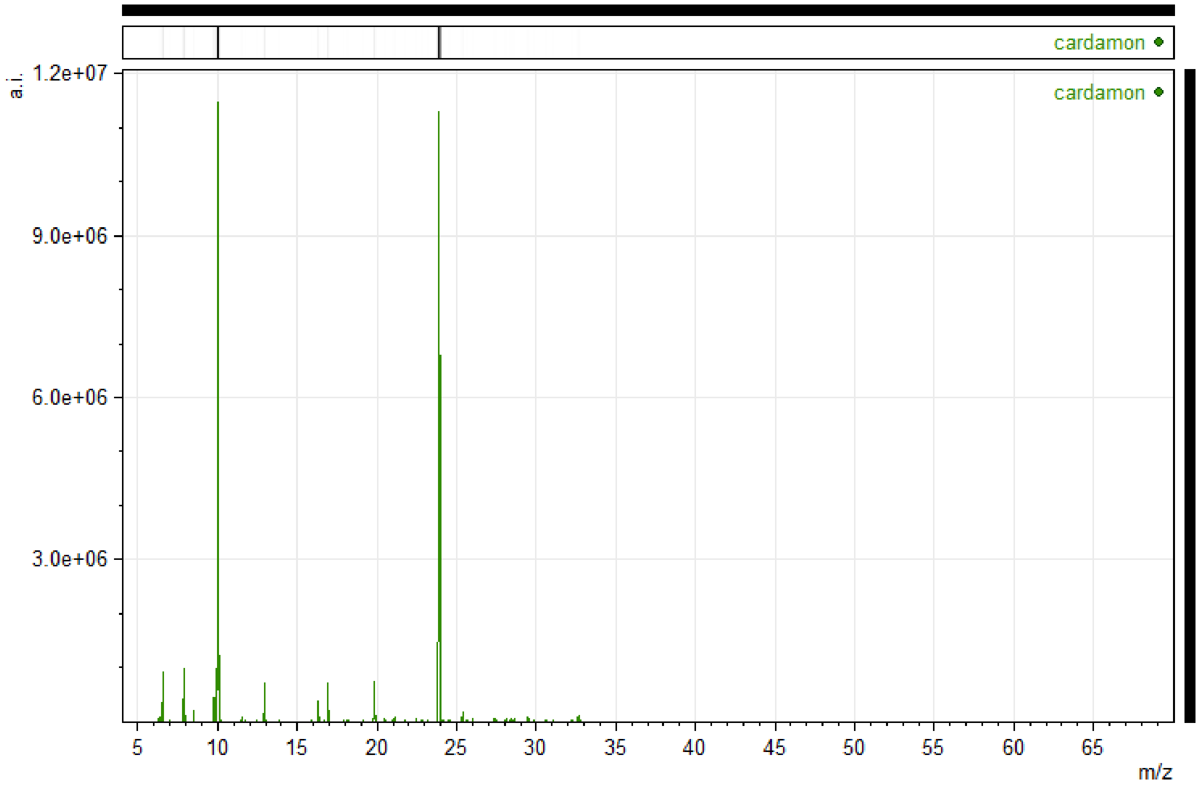

Figure S7. GC-MS FID chromatographic characteristics of cardamon (*Pelargonium graveolens* L.) composition visualized in mMass software.

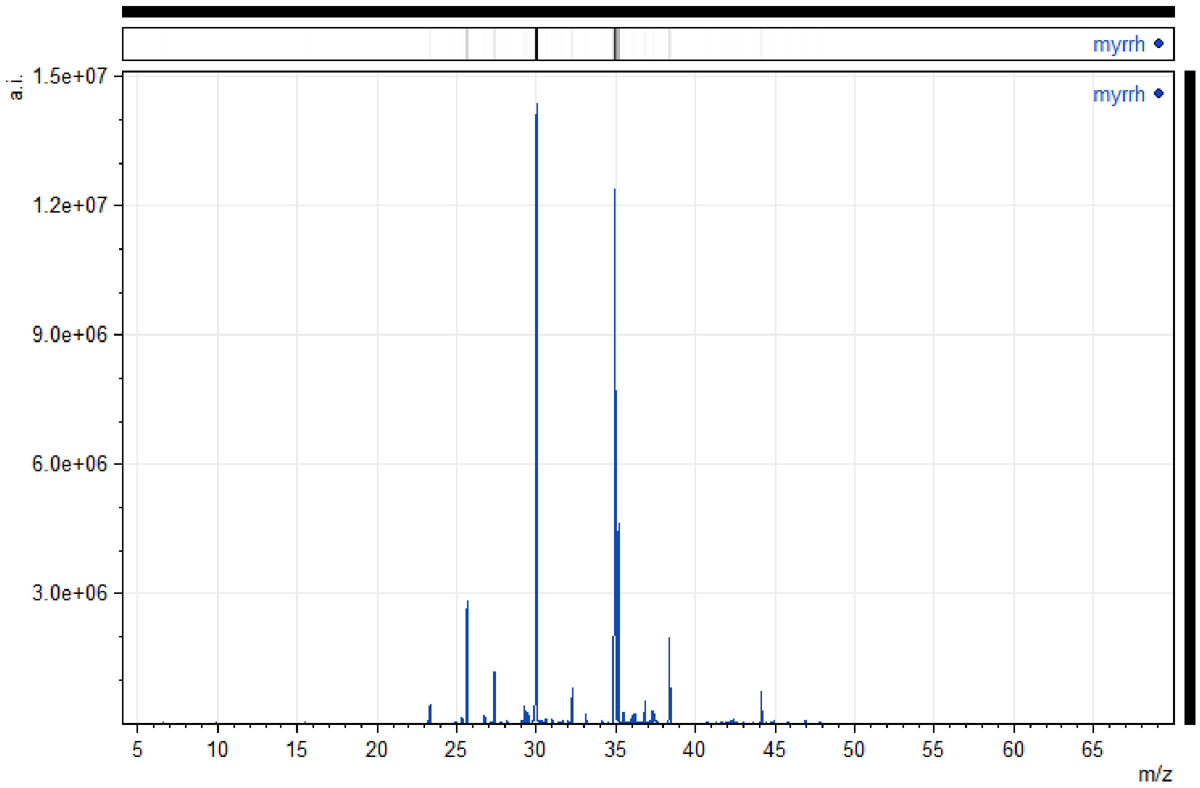

Figure S8. GC-MS FID chromatographic characteristics of myrrha (*Commiphora myrrha* Nees) composition visualized in mMass software.

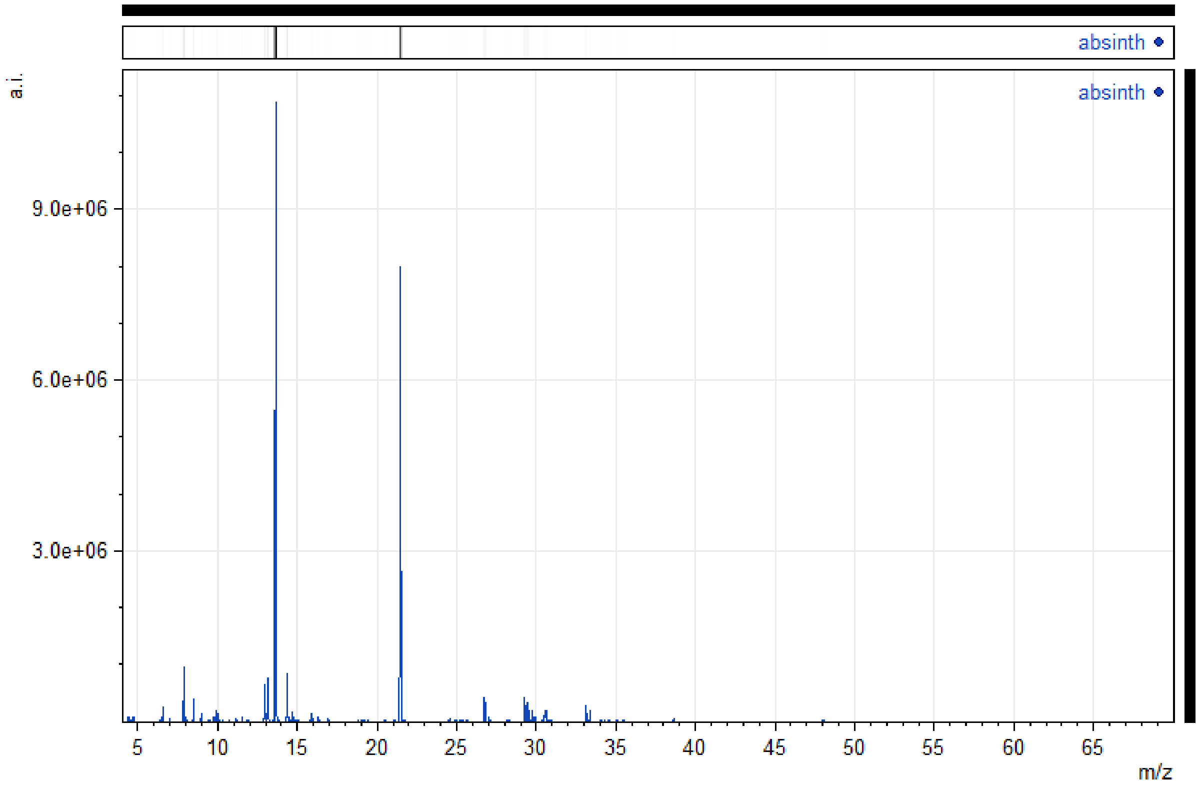

Figure S9. GC-MS FID chromatographic characteristics of absinth (*Artemisia absinthium* L.) composition visualized in mMass software.

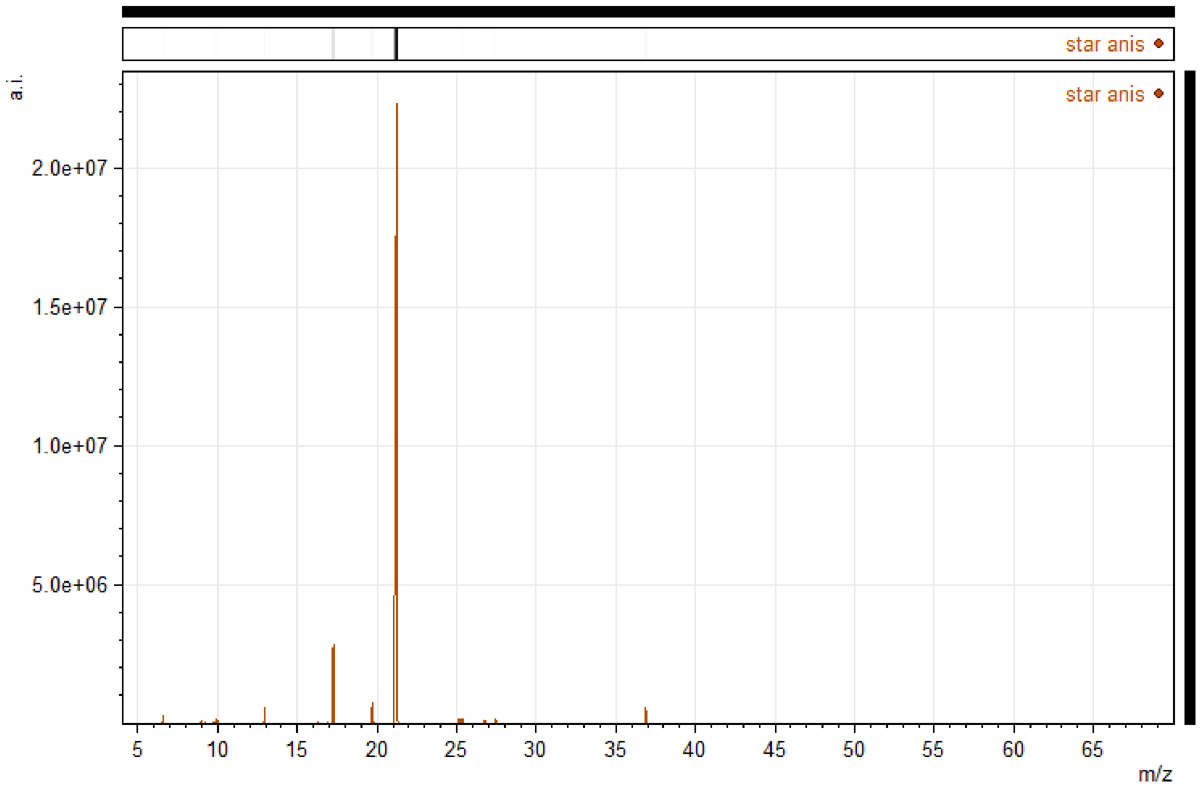

Figure S10. GC-MS FID chromatographic characteristics of star anise (*Illicium verum* Hook. f.) composition visualized in mMass software.

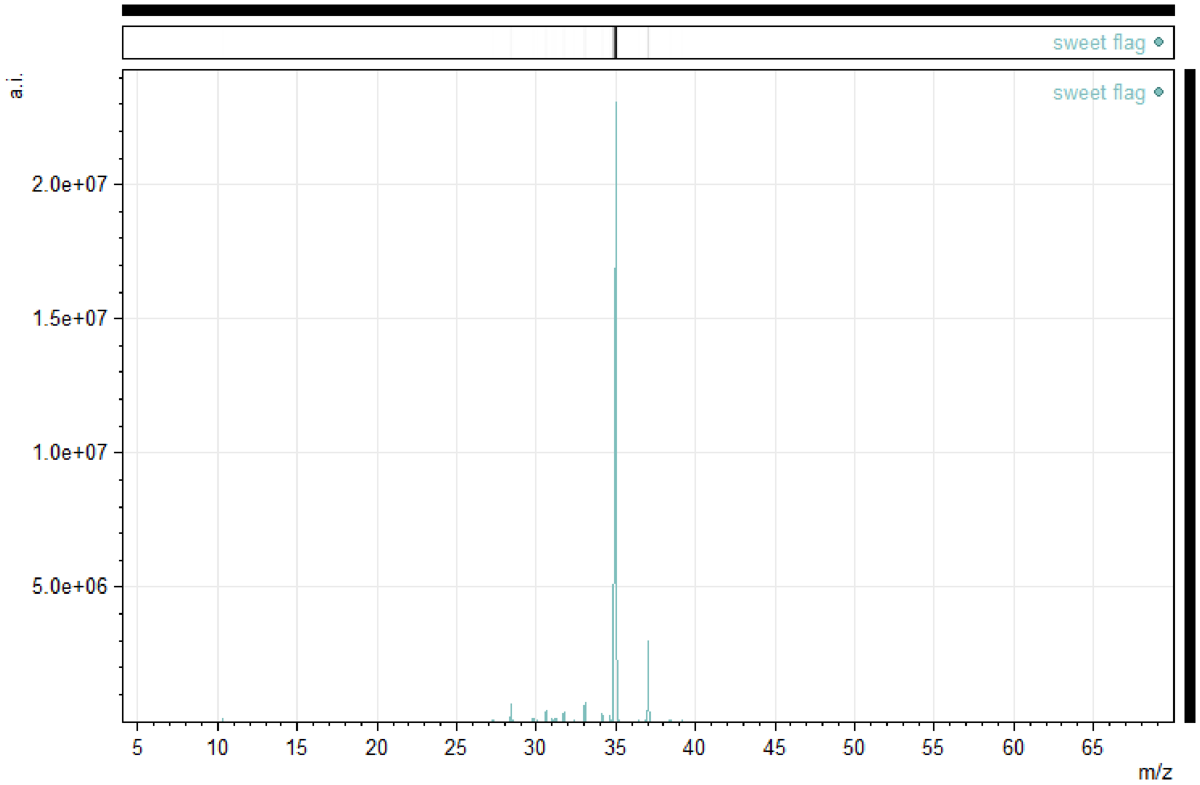

Figure S11. GC-MS FID chromatographic characteristics of sweet flag (*Acorus calamus* L.) composition visualized in mMass software.

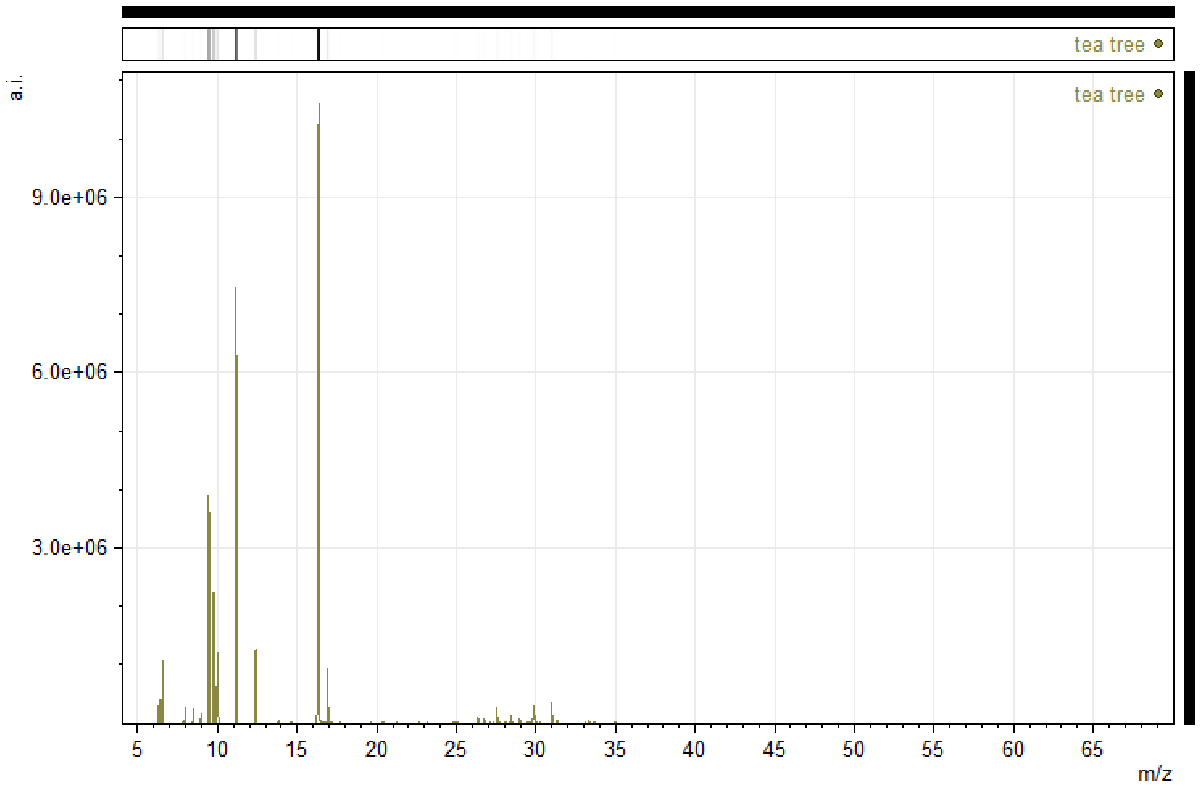

Figure S12. GC-MS FID chromatographic characteristics of tea tree (*Melaleuca alternifolia* L.) composition visualized in mMass software.
